# The enhancement of cartilage regeneration by use of a chitosan-based scaffold in a 3D model of microfracture in vitro: a pilot evaluation

**DOI:** 10.1186/s40634-021-00328-z

**Published:** 2021-02-18

**Authors:** N. Andjelkov, H. Riyadh, M. Ivarsson, Z. Kacarevic-Popovic, J. Krstic, P. Wretenberg

**Affiliations:** 1 Department of Orthopedics, Västmanlands Regional Hospital, Västerås, Sweden; 2 Centre for Clinical Research, Uppsala University, Västmanlands Regional Hospital, Västerås, Sweden; 3 Department of Orthopaedics, School of Medical Sciences, Örebro University, Örebro, Sweden; 4 Department of Health Sciences, University of Örebro, Örebro, Sweden; 5 Department of Radiation Chemistry and Physics, Vinca Institute of Nuclear Sciences, University of Belgrade, Belgrade, Serbia

## Introduction

Even though microfracture as a technique has been in use for a long time [29], a discussion about the exact mechanism for cartilage repair during this technique and cartilage repair techniques derived from it is still open [10]. This technique will not be more thoroughly described here since it is the best known and most widely used cartilage repair procedure at the present. What’s important to mention here is that it’s based on the bone marrow stimulation using mesenchymal stromal cells (MSCs) from the subchondral bone for regeneration [29]. A variety of matrix-assisted microfracture techniques have been in use since the original technique was developed [9, 22]. The AMIC (Assisted Matrix Induced Chondrogenesis) technique is one of those scaffold-based techniques which was derived from the microfracture [21]. It has been known that these techniques may lead to the creation of hyaline and fibrocartilage [11] but the knowledge about what exactly happens on the cellular and tissue level during the repair process itself is still insufficient.

The main goals of the study were to detect the basic processes like cell migration and differentiation, extracellular matrix formation and expression of some crucial bio-markers of chondrogenesis depending on different experimental conditions – absence or presence of three different types of scaffolds, i.e. fibrin, collagen-based and chitosan-based scaffold. The method used was procollagen type I, procollagen type II, and SOX-9 detection as one of the crucial biomarkers of fibrocartilage, hyaline cartilage and chondrogenesis respectively [3, 30]. Gomori’s staining for detection of extracellular cartilage matrix formation has also been used [8]. The main study hypothesis was that the use of the scaffolds would improve all the parameters mentioned above.

## Materials and methods

**Fig. 1:**
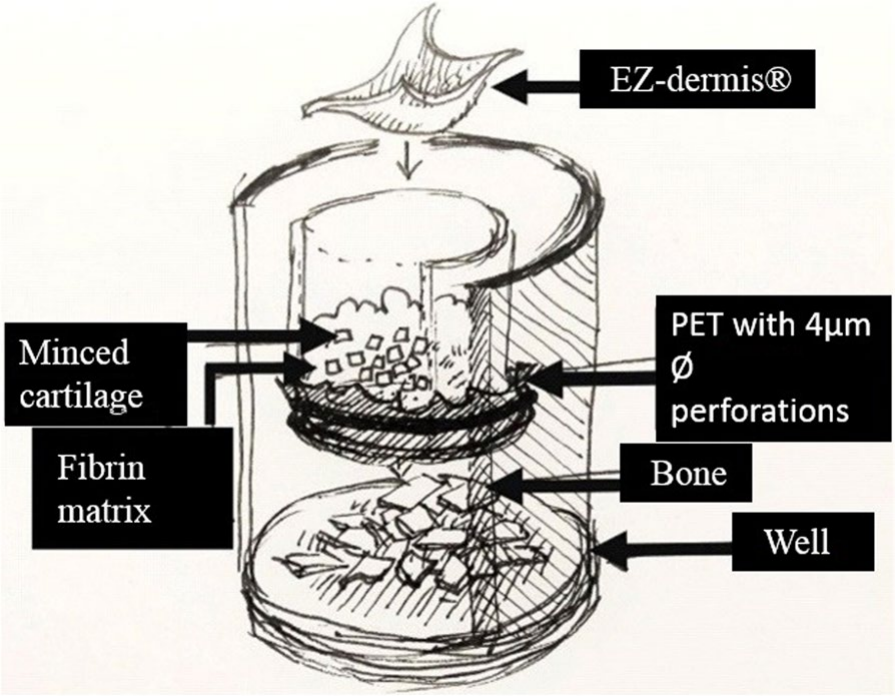
Microfracture 3D – model used in our experimental setup in vitro: EZ Derm® – porcine epidermis. Minced cartilage. Fibrin matrix – in some groups it has been replaced with chitosan-based matrix. PET membrane with 4 µm pores and perforated with BD Microlance® 21 g × 1"—0,8 × 25 mm needle. Bone chips at the bottom of plastic well

A 3-D model in vitro that should represent the microenvironment that is created by microfracturing (Fig. 1) was made and the cells were cultured for eight weeks in different experimental conditions. Subsequently, the constructs containing a 3-D model were removed from the cell culture medium, fixed in paraffin and analyzed with immunohistochemistry.

### Culture of cartilage in a 3-D model

Cartilage and trabecular bone were obtained from the discarded femoral heads during hip arthroplasty (two patients, both women, Pat. No. 1, 70 years of age, Pat. No 2, 69 years of age, both with osteoarthritis grade II-IV), as well as from the patients undergoing total knee replacement surgery, where macroscopically normal cartilage could be found in the lateral femoral condyle area (two patients, both women, Pat. No. 3, 76 years of age, Pat. No 4, 75 years of age). The specimens from all four patients were collected and the experiments were repeated for each of them. The cartilage was cut into small pieces (1–2 mm^3^) and the trabecular bone was cut into bone chips small enough to fit the wells and the space under the inserts, and then incubated in different experimental settings according to Table 1, some of them without and some of them with 20 units/ml collagenase II, respectively 100 units/ml collagenase II (Sigma-Aldrich, Missouri, USA) in Dulbecco's modified eagle medium (DMEM) nutrient solution (ThermoFisher, Waltham, Massachusetts, USA) at 37 °C for 16 h under gentle movement. The samples were centrifuged at 260xg for five minutes and washed in 10 ml phosphate buffered saline (PBS) (ThermoFisher, Waltham, Massachusetts, USA) followed by centrifuged for 260xg for five minutes. The final pellet was then placed in a special insert containing 8-μm PET or 1.2-μm nylon membrane (Fig. 1; CellCrown™24, Cat. Nr. C70001F, C12001F, Scaffdex, Tampere, Finland) perforated with a sterile 0.6 × 25-mm needle. In this model, this upper compartment should represent the cartilage defect that could be found in microfracture after debridement of cartilage injury. The needle perforations through the membranes in the bottom of the inserts represented the holes that are made in the subchondral bone during microfracture. The fibrin matrix, replacing natural fibrin that could be found in the “super clot” [29] post microfracture (TISSEEL DUO QUICK, Baxter, Sweden) was then added covering in total cartilage fragments, and the inserts were placed onto the top of the cancellous bone biopsies previously situated in a sterile 24-well plate. In this model, the cancellous bone chips represented the subchondral bone in microfracture and could easily be separated from the inserts at the end of cell culturing and prior to inserts analysis. Finally, some inserts were left without any scaffolds, in some of them as previously mentioned, fibrin was added, while some inserts were filled with chitosan-based scaffold made by this research group and some were covered with EZ Derm® – porcine skin consisting of a superficial thin layer mainly made of dead cells – epidermis, and another much thicker layer – dermis consisting mainly of collagen fibers, which should replace commercially available collagen-based scaffolds currently used in certain cartilage repair techniques. Wells were filled with DMEM supplemented with 20% fetal bovine serum (FBS) and antibiotics, so called “growth medium” that has been changed after a week with a DMEM supplemented with 10% serum. The nutrient solution was changed twice a week for eight weeks with DMEM and 10% FBS. An overview of the contents of the wells is shown in Table 1.

**Table 1:** Eight weeks of culture in CellCrown®. Perforated PET membrane in all groups. Some groups weren’t pretreated with collagenase while the others were predigested with both low and high enzyme concentrations. Some groups didn’t contain any scaffold, some groups contained fibrin, some groups EZ-derm®, while other groups contained chitosan-based scaffold. Upper compartment with PET membrane was fixated, emerged in paraffin and histology sections were made. Hematoxylin, eosin and trichrome staining was done. Immunohistochemistry analysis for four markers was performed: procollagen type I and II, SOX-9 and S-100

Well	Plate	Bone	Cartilage	Collagenase (conc.)	Fibrin	EZ-derm	Chitosan
1	A	+	+	-	-	-	+
2	A	+	+	-	-	+	-
3	A	+	+	-	-	+	-
4	A	+	+	-	+	+	-
5	A	+	+	-	-	+	-
6	A	+	+	-	+	+	-
7	A	+	+	-	-	+	-
8	B	+	+	Low	-	-	+
9	B	+	+	Low	-	+	-
10	B	+	+	Low	+	+	-
11	B	+	+	Low	-	+	-
12	B	+	+	Low	+	+	-
13	B	+	+	Low	-	+	-
14	B	+	+	High	-	-	+
15	B	+	+	High	-	+	-
16	B	+	+	High	+	+	-
17	B	+	+	High	-	+	-
18	B	+	+	High	+	+	-
19	B	+	+	High	-	+	-
20	B	-	+	High	-	+	-
21	A	+	-	-	-	+	-

### Fixation and dehydration of cultures

After 8 weeks of culture, the content of the inserts was taken out and positioned in padded cassettes so that central portions were facing surface after paraffin embedding. Specimens were fixed in 3.7% formaldehyde. Dehydration was performed with Tissue Processor TPC15DUO (Medite GmbH, Burgdorf, Germany).

### Embedding and sectioning

After dehydration, fixed specimens were embedded in paraffin. Four micrometer tissue sections were generated with Leica RM2255 automatic microtome (Leica biosystems, Mölndal, Sweden). The sections were floated in a cold-water bath and mounted on Super frost® plus gold microscope glass-slides (Fisher Scientific, Gothenburg, Sweden). Slides were placed on a hot plate for one minute. Slides were placed in a 60 °C heating cabinet for four days.

### Hematoxylin and eosin staining

The sections were rehydrated in xylene/ ethanol/water, stained with Mayer’s hematoxylin (Histolab, Gothenburg, Sweden) for five minutes and rinsed with tap water for four minutes. After that, they were stained for one minute in eosin solution (distilled water, 0.25% eosin, 80% ethanol, and 90 mM acetic acid) and rinsed with tap water for one minute. Finally, the sections were dehydrated in distilled water/ethanol/xylene and mounted with cover slips and Pertex mounting agent (Histolab, Gothenburg, Sweden).

### Gomori’s trichrome staining

Rehydrated sections were stained with Harris hematoxylin for six minutes and rinsed for five minutes with tap water. Sections were stained with Gomori’s stain solution A (0.6% chromotrope 2R, 0.6% phosphovolvic acid hydrate, 0.3% Fast Green FCF, 0.17 M acetic acid and distilled water) for ten minutes and rinsed with distilled water. Slides were briefly immersed in Gomori's stain solution B (0.35 M acetic acid and distillate water), dehydrated and mounted.

### Immunohistochemistry

Immunohistochemistry staining was performed with Dako REAL ™ EnVision ™ Detection System Peroxidase / DAB + , Rabbit / Mouse (Dako, Agilent, Santa Clara, California, USA). Rehydrated sections were washed twice in wash buffer and incubated with Proteinase K for ten minutes. Thereafter, they were rinsed twice with wash buffer and incubated with primary antibodies S100 (ThermoFisher, Waltham, Massachusetts, USA), procollagen II (Abcam, Cambridge, UK) or Sox9 (Abcam, Cambridge, UK), diluted 1: 500 for 30 min. The sections were rinsed then with wash buffer twice and incubated with peroxidase blocking for eight minutes. Next, the sections were rinsed with wash buffer twice and incubated with EnVision complex for 30 min. Sections were rinsed twice with washed buffer and incubated with Chromium (DAB) for 15 min. Sections were rinsed with wash buffer twice and rinsed in running tap water for 5 min. Finally, they were stained with Mayer's hematoxylin for ten seconds and rinsed with tap water for five minutes. After dehydration, slides were mounted with Pertex mounting agent.

## Results

### Culture of cartilage in a 3-D model

Bone marrow—derived cells migrated to the upper compartment of the construct through a perforated nylon membrane containing either enzymatically digested- or non-digested particulated cartilage. If not predigested with collagenase, the chondrocytes weren’t able to escape the matrix and remained within the cartilage pieces.

### Hematoxylin and eosin staining

**Fig. 2:**
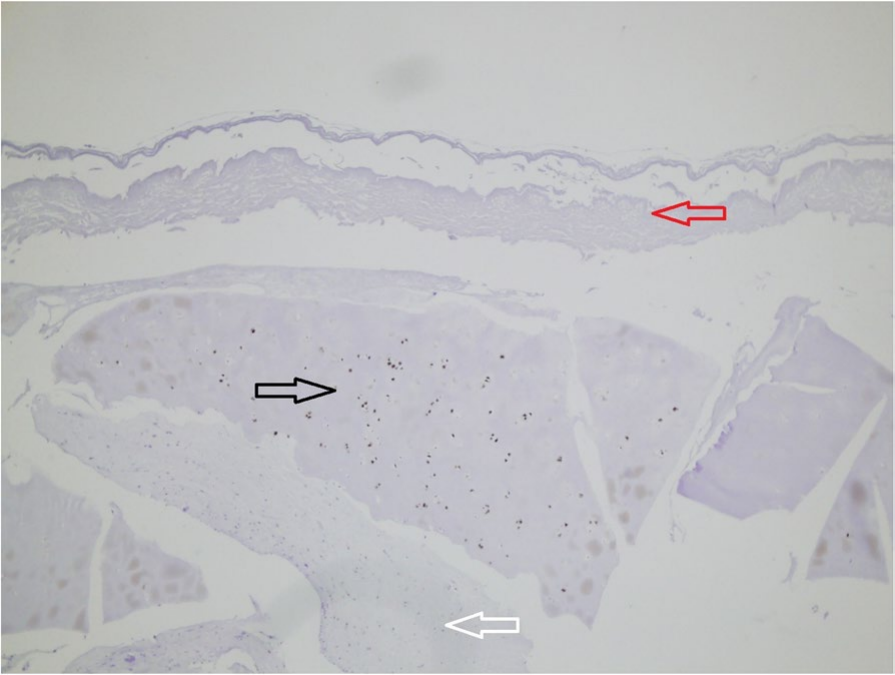
Minced cartilage + bone chips + fibrin, no digestion with collagenase (Well 4): Positive staining with S-100 for chondrocytes situated in the lacunas, no chondrocytes seen outside the cartilage pieces (marked with black arrow). Cell elements with extracellular matrix beneath the cartilage piece positioned centrally in the figure. These cells weren`t stained with S-100 and supposedly originate from the bone chips, i.e. bone-marrow derived cells (marked with white arrow). In the upper corner porcine epidermis with no cell elements (marked with red arrow) can be seen. No adhesion of the newly formed matrix with the piece of cartilage

Bone-marrow derived cells have migrated towards minced cartilage (Fig. 2), New, extracellular matrix was created outside the non-digested cartilage pieces (Fig. 2). Without cellular elements in the upper compartment, i.e. chondrocytes, but only EZ-derm, no bone-marrow derived cells migration were noticed towards or through the membrane.

### Gomori’s trichrome staining

**Fig. 3:**
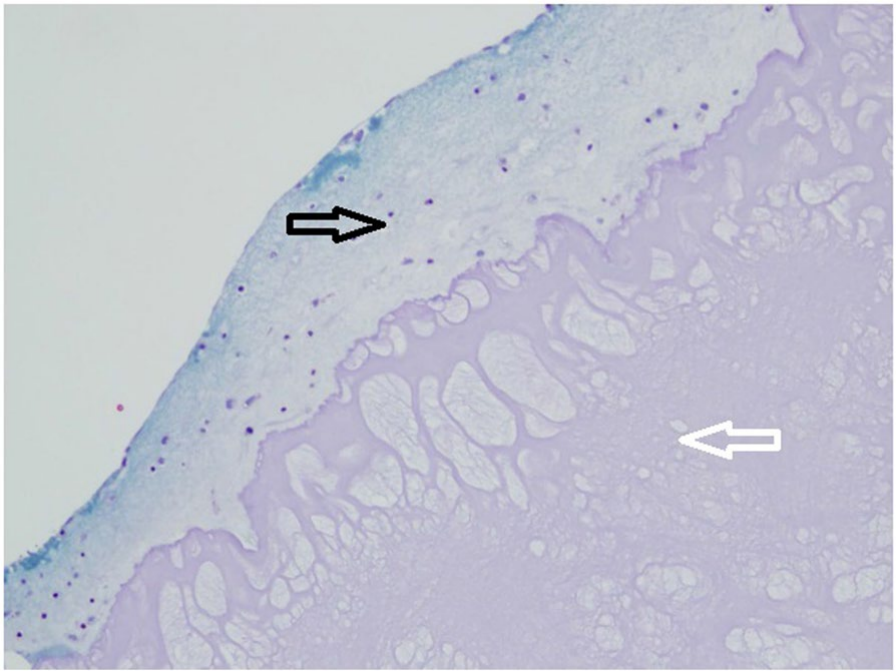
New cartilage formation in the upper part of the photo detected with Gomori staining and visualized in blue (marked with black arrow, well 8). The cell elements situated within the newly formed cartilage matrix. Chitosan based scaffold served as a matrix in the lower compartment stained in pink (marked with white arrow). God adhesion of the newly formed extracellular matrix to the scaffold

A collagen-specific extracellular matrix next to the chitosan-based scaffold was confirmed with trichrome (Gomori) staining (Fig. 3). The attempts to show positive staining with trichrome failed in all other experimental groups with the exception of non-digested cartilage pieces, which were stained as expected.

### Immunohistochemistry

**Fig. 4:**
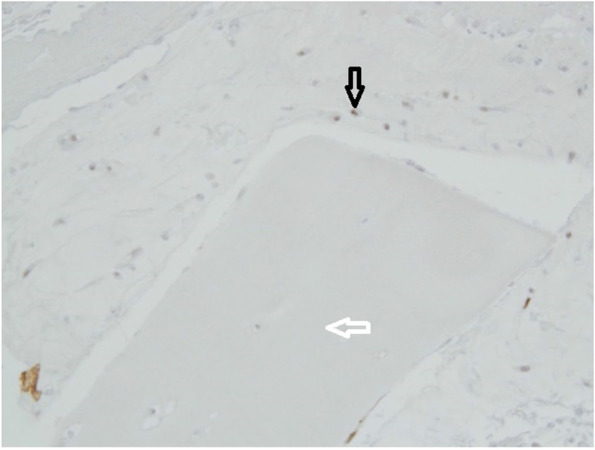
Some week positive signals for SOX-9 stained cells in the newly formed cartilage matrix from the digested particulated cartilage (marked with black arrow, well 10). Minced cartilage in the middle (marked with white arrow). Poor adhesion of the newly formed matrix with the piece of cartilage

**Fig. 5:**
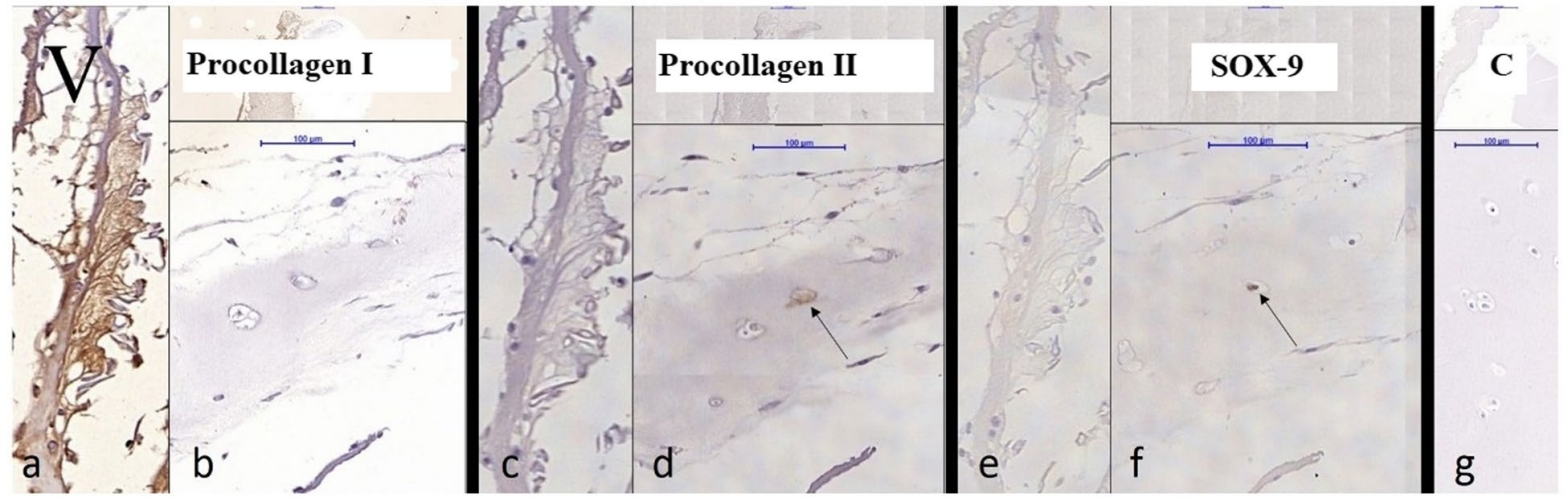
Cartilage cultivation containing cartilage pieces treated with 100 U/ml collagenase and cultured with fibrin matrix (Well 16). **a** and **b**: Fibrin respectively cartilage. Figure a representing immunohistochemistry with antibody against procollagen type I that was non-specific and **b** representing positive staining within cartilage. **c** and **d**: Fibrin respectively cartilage. Negative respectively positive immunohistochemistry with antibody against procollagen type II. **e** and **f**: Fibrin respectively cartilage. Negative respectively positive immunochemistry with antibody against sex-determining region Y-related high-mobility-group box 9 (SOX-9). **g**: Negative control for SOX-9

**Fig. 6:**
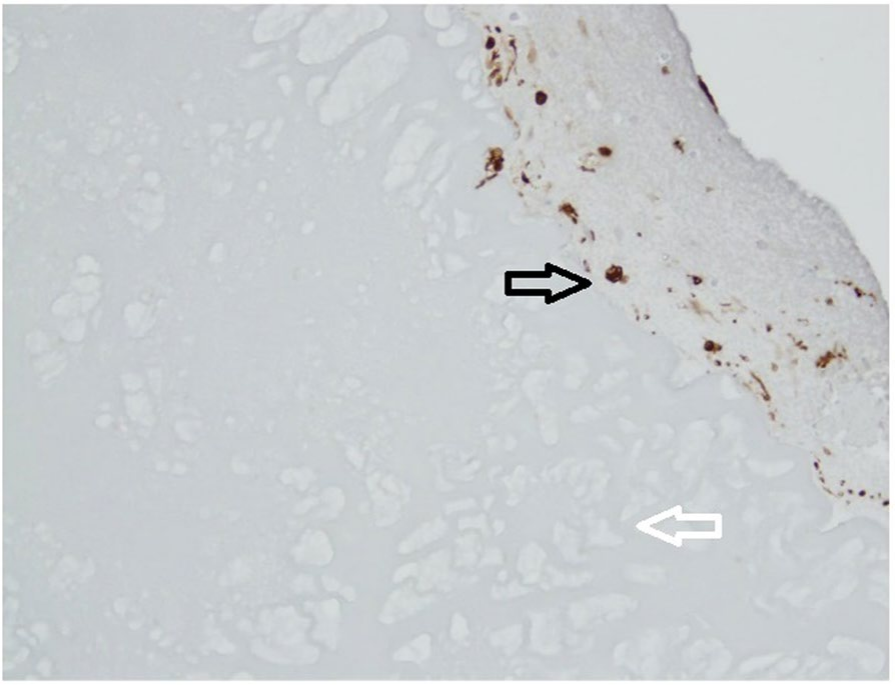
Immunohistochemistry with S-100 (Well 8). Scaffold in the lower corner (marked with white arrow), newly formed cartilage matrix in the upper corner (marked with black arrow). Sporadic staining with S-100 at the periphery of the newly formed cartilage matrix and more abundant signals in the newly formed cartilage next to the scaffold (marked with black arrow). God adhesion of the newly formed extracellular matrix to the scaffold

Chondrocytes were identified by staining with S-100 and bone derived cells were remained unstained (Fig. 2). Bone-derived cells and not chondrocytes have created new extracellular matrix in the groups without cartilage digestion as showed by S-100 staining (Fig. 2). When predigested with collagenase, chondrocytes from minced cartilage have formed new cartilage matrix as well (Fig. 4). SOX-9 staining was proved to be positive both inside the cartilage pieces (Fig. 5) and with less intensity in the newly created cartilage matrix (Fig. 4). In the last case, chondrocytes within the non-digested cartilage pieces remained unstained with SOX-9 (Fig. 4). Staining with procollagen type II was positive inside of minced cartilage (Fig. 6d), but not outside of it (Fig. 5c). The antibody for procollagen type I gave a strong non-specific staining, the reason why it couldn’t be used for further analysis (Fig. 5a and b).

## Discussion

The main findings of this study are the observations related to the use of different type of scaffolds on basic processes like cell migration, differentiation and creation of new cartilage matrix. We have seen in this study the differences in these important processes depending on the absence of any scaffold or the use of various type of scaffolds, namely fibrin, collagen-based or/and chitosan-based scaffold. At present, there are numerous reports of the improvement of cartilage repair with the use of various types of scaffolds [2, 22], while other reports show no such effect [12, 16]. Most of those studies have been conducted as clinical trials [16, 22], giving us the objective measures such as histology and clinical scores, which naturally is of greatest interest. Numerous animal studies have been conducted as well, prior to the use of these scaffolds in humans [12, 32], providing us with the histology of the repaired tissue when using different scaffolds or without them [12, 32]. However, the studies on the basic level, preceding both animal models and clinical trials are not abundant and the authors of this article have failed to find them in the literature. Therefore, this 3-D model in vitro of microfracture, presented here can be seen as an attempt to add some new knowledge to this topic and would hopefully lead to the improvement of the scaffolds used in matrix-assisted microfracture techniques prior to their clinical use and subsequently the improvement of clinical outcomes as well.

### Scaffold-free experimental setup

The most relevant observation when not having any scaffold in the upper compartment has been that cellular migration towards and within upper compartment hasn't been noticed. This finding is somehow expected since it has been known that the fibroblast-like cells that are phenotypically similar to MSCs [7] when in culture may need chemo-tactical, mechano-tactical and electro-tactical factors for cell migration [6, 25]. This would be one of the main limitations of this model since it has been known that even without the scaffold, the space within debrided cartilage is almost immediately filled with blood, growth factors and fibrin, a so called “super clot” formation [29].

Another limitation of this model that should be mentioned and discussed at this point is the use of collagenase in certain experimental groups. In this 3-D model of microfracture in vitro bone-derived cells and not chondrocytes have shown to have an active role in new cartilage formation when minced cartilage haven’t been pretreated with collagenase. However, when digested with collagenase, chondrocytes alone from particulated cartilage have created a neocartilage formation. Under normal circumstances, the predigestion with collagenase during microfracture procedure doesn’t occur. Since it has been known from a previous study from this group [1] that the chondrocytes wouldn’t be able to escape the matrix without the enzymatic digestion, the collagenase has been used. This finding has been confirmed even in this study since no outgrowth of chondrocytes has been noticed in the non-digested experimental groups (Fig. 2). However, even in a normal, clinical situation in microfrature, a smaller amount of proteolytic enzymes may be present at the site of injury and inflammation [17, 28]. Nonetheless, this concentration is unlikely to be in such high concentrations as those used in the experiments and therefore it doesn’t seem likely that chondrocyte relase occurs in microfracture. Consequently, the probability that in a clinical situation during microfracture the enzymatic digestion of cartilage takes place so that “released” chondrocytes can participate actively in the novel cartilage matrix synthesis is very low. Finally, no significant differences have been found for 20 units/ml collagenase II, respectively 100 units/ml collagenase II.

### Fibrin scaffold

Fibrin has been used widely in cartilage repair procedures both alone as a scaffold [31] or as a glue to secure the scaffold in place post surgery [24]. In the experimental model applied in this study, it hasn't been seen any migration of the cells into the fibrin matrix and therefore no new extracellular matrix formation inside. Similarly, very poor or no integration to other scaffolds and extracellular matrices has been noticed (Figs. 2 and 4). A commercially available fibrin preparation has been used in this study, very similar to those routinely used for matrix fixation during matrix-assisted microfracture techniques [27]. Despite the mechanical equivalency, this fibrin preparation differs in some characteristics from the autologous one that may be present in the normal clinical situation in microfracture [14].

### Collagen-based scaffold

As for the collagen-based matrices, the explanation has already been given in the “Materials and Methods” section for the choice of use of EZ-derm® as a substitute for a collagen-based matrix. Indeed, this type of scaffolds has been widely used in so-called matrix-assisted microfracture techniques [16]. By its structure, the EZ-derm® is almost identical to the one of most commonly used matrices in cartilage repair which has also been derived from the porcine skin – Chondro- Gide® [27]. In the histological sections obtained in this study, scarce or no cell migration has been seen towards or into the EZ-derm® (Figs. 1 and 2). Subsequently, no extracellular matrix production has been identified next to it (Fig. 2). The poor integration of EZ-derm® with the other components of the construct has been seen as well (Fig. 2). In this experimental group, the new cartilage matrix has been synthesized separately and beneath the EZ-derm® (Fig. 2). As previously mentioned, chondrocytes within minced cartilage have been stained with S-100 (Fig. 2) as a rule. The staining with procollagen type II has been positive within minced cartilage and so far, hasn’t been detected outside of it, in the newly formed extracellular matrix to be more specific (Fig. 5c and d). At the present, the conclusion that can be made from these findings is that the chondrocytes from minced cartilage are metabolically active and are still producing new collagen type II after 8 weeks in culture [4, 13]. As said previously, the staining with procollagen type I was unspecific and therefore these results can´t be used for any further analysis (Fig. 5a and b). Future immunohistochemistry for both procollagen type I and II is recommended for better understanding of their expression dynamics and distribution in newly created extracellular matrix. This with concern to the fact that collagen type I is primarily expressed in fibrocartilage [19] while collagen type II is the crucial marker of hyaline-like cartilage [33]. That is why it is very important to further examine the expression of these two biomarkers, specifically in relation to their presence in scaffolds or not. The staining with SOX-9 has mainly been detected in cartilage pieces (Fig. 5f). At the same time, weak SOX-9 staining has been noticed in the newly formed cartilage matrix (Fig. 4). This matrix has been synthetized from the chondrocytes originated from digested particulated cartilage. According to the previous reports the SOX-9 gene is expressed from the multipotent skeletal progenitor stage and is active throughout chondrocyte differentiation [18]. It is repressed in hypertrophic chondrocytes in cartilage growth plates but remains expressed throughout life in permanent chondrocytes of healthy articular cartilage [18]. The absence of SOX-9 expression in matrix in most of the cases therefore suggests the presence of cells other than chondrocytes in these sections, most probably bone-marrow derived cells. Reportedly, MSCs become hypertrophic in long term despite chondrogenic differentiation following the pathway of growth plate chondrocytes [26]. All together, by failing to prove both the markers of chondrogenesis (SOX-9, procollagen type I and II), the marker of adult chondrocyte phenotype (S-100) in the cell elements within the newly synthetized extracellular matrix, and cartilage-like nature of that matrix (trichrome/Gomori staining), the chondrogenic potential of the collagen-based scaffold in this experimental model hasn't been confirmed. In addition to that, as previously said very poor integration with other components of the insert, as well as scarce cell adhesion have been detected.

### Chitosan-based scaffold

Chitosan has been routinely used as a scaffold in cartilage repair [22]. Some very good clinical data have been reported on its use [22]. In this model, similarly as in the case of collagen-based scaffolds, a substitute to the commercially available scaffold has been used. This time, the scaffold used was made by this research group. At this point, a further description of the scaffold characteristics is unfortunately not possible due to patent rights protection.

First of all, an excellent integration of the newly formed cartilage matrix, as proved by Gomori’s staining, has been seen (Fig. 3). These two findings speak strongly in favour of the chondrogenic potential of this scaffold. However, only certain cells in the newly formed cartilage matrix have been stained with S-100. These were mainly situated next to the chitosan-based scaffold (Fig. 6). From past studies it has been known that S-100 is expressed in differentiated MSCs but not in an undifferentiated cell form [5]. One explanation could be that chondrocytes have been detected in the newly created extracellular matrix. The other possible explanation could be that not all chondrocytes express S-100 as it has been reported before [20]. In this study, the immunostaining for both S-100 sub units (a and b) was detected in chondrocytes in superficial, intermediate, and deep zones of normal articular cartilage [20]. However, in matrix, only the superficial zone was stained positively [20] In arthritic joints, intense immunostaining was detected in clustered chondrocytes in the hypercellular area, while weak or no immunostaining have been seen in isolated chondrocytes in the hypocellular area of articular cartilage [20].

No staining with SOX-9 has been detected next to the chitosan-based scaffold. SOX-9 is a transcription factor that regulates chondrogenesis and its role in chondrogenic differentiation of MSCs triggered by materials is poorly understood [15]. A study has shown that the role of SOX-9 regulation by materials is like that of growth factors, suggesting that a well-designed scaffold may replace growth factors in chondrogenesis [15]. A correlation between cell viability and SOX-9 downregulation has also been reported [23]. Probably, additional experiments should be done at different times to look for the expression of this and other bio-markers. This could be one of the main flows of this study.

Finally, similar to the case of other scaffolds no or very poor migration of the cells into the depth of the matrix has been seen. However, the preliminary data that hasn't been reported in this study shows scarce cell migration into this matrix by identifying cellular DNA inside of the matrix. Again, not going into details about the matrix structure, the pore size should be enlarged to increase the permeability of the matrix for the cells.

This 3-D model of microfracture in vitro may be seen as a very useful tool for the analysis of the basic cell processes in microfracture and the influence of different scaffolds on the processes leading to cartilage repair. Furthermore, it could serve as a suitable environment for making modifications and improvements in scaffolds prior to their clinical use in the matrix-assisted microfrature techniques. The authors of this text consider that as the main clinical relevance and contribution of this 3-D´model. Further studies should be conducted for better understanding of the dynamics of cartilage bio-markers expression in microfracture and the influence of different types of scaffolds and their adjustments on this expression and neocartilage formation.
